# Safety of endoscopic mucosal resection (EMR) of large non-pedunculated colorectal adenomas in the elderly

**DOI:** 10.1007/s00384-017-2892-7

**Published:** 2017-09-08

**Authors:** K. Bronsgeest, J. F. Huisman, A. Langers, J. J. Boonstra, B. E. Schenk, W. H. de Vos tot Nederveen Cappel, H. F. A. Vasen, J. C. H. Hardwick

**Affiliations:** 1 Department of Gastroenterology and Hepathology, Leiden University Medical Centre, Albinusdreef 2, 2333 ZA Leiden, The Netherlands; 2 Department of Gastroenterology and Hepathology, Isala, Zwolle, The Netherlands

**Keywords:** Endoscopic mucosal resection, EMR, Colorectal adenomas, Non-pedunculated adenomas, Giant adenomas, Complications, Bleeding, Perforations, Elderly

## Abstract

**Background:**

Endoscopic mucosal resection (EMR) has been proven to be safe and effective for the treatment of colorectal adenomas. However, data are limited on the safety of this technique for large polyps and in elderly patients. Aims of our study were to examine the bleeding and perforation rates in patients with large non-pedunculated adenomas (≥20mm) and to evaluate the influence of size (≥40mm) and age (≥75 years) on the complication rates.

**Methods:**

In this multicenter retrospective study, patients who underwent EMR of non-pedunculated adenomas ≥20mm between January 2012 and March 2016 were included. The demographics of the patients, the use of antithrombotic drugs, size of the polyps, type of resection, pathology report, occurrence of post-polypectomy bleeding, and perforation- and recurrence rate were collected.

**Results:**

In 343 patients, 412 adenomas were removed. Eighty patients (23.3%) were ≥75 years of age, 138 polyps (33.5%) were ≥40mm. Bleeding complications were observed in 28 cases (6.8%) and were found significantly more frequent in adenomas ≥40mm, independent of the use of antithrombotic therapy. Five perforations (1.2%) were described, not related to the size of the polyp. There was no significant difference in complication rates between patients <75 years and patients ≥75 years. Bleeding complications rates were significantly higher in patients receiving double antithrombotic therapy.

**Conclusion:**

EMR is safe in elderly patients. EMR of adenomas of ≥40mm was associated with more bleeding complications. Future studies should address how the bleeding rates can be reduced in these patients, especially in those who use double antithrombotic treatment.

## Introduction

Colorectal cancer is the most commonly diagnosed cancer of the gastro-intestinal tract. It is the leading cause of overall cancer death in Western countries after lung cancer [1–7]. In 2012 in Europe, the incidence of colorectal cancer was 60 per 100.000 people, resulting in nearly half a million new cases per year. The mortality rate of 29 per 100.000 accounted for 12.2% of all cancer deaths. The incidence of colorectal cancer is increasing and it is expected to rise from 1.4 to 2.4 million cases annually worldwide by 2035 [1, 2].

In many countries, population screening programs for the detection of colorectal cancer have been implemented, usually starting at the age of 50 or 55 and continuing until the age of 75 [5–8]. Screening tests including fecal occult blood test (FOBT), sigmoidoscopy or colonoscopy are used to detect early stage colorectal cancer and are proven effective in reducing mortality and morbidity rates [1, 5–7]. Such programs lead to the detection of an increasing number of patients with large adenomas [9]. Critics of the colorectal cancer screening programs point towards the lack of evidence for a decrease in overall mortality, possibly due to the advanced age and extensive comorbidity in those found with colorectal cancer. Detection and removal of large polyps in this frail patient group has the potential to lead to morbidity and mortality that could negate any positive effects of the screening [10]. To be able to balance the health benefits with the risks, more information as to the nature and extent of these risks in this subgroup is required.

For the removal of large non-pedunculated polyps, endoscopic mucosal resection (EMR) is the usual treatment, and is reported to be effective and safe [11–31]. However, in polyps of ≥20mm, piecemeal resection is often required which is associated with higher recurrence rates [11, 15–17, 20, 32, 33].

The prevalence and size of colorectal polyps increases with age, so an increasing number of patients over the age of 75 are likely to undergo EMR. Various studies have reported on the complication rate of endoscopic resection in non-pedunculated polyps ≥20mm [11, 14, 19, 27, 28, 34]. However, information about the complication rate of endoscopic resection of giant polyps (≥40mm) and in elderly patients (≥75 years) is limited [26, 27, 34, 35]. Therefore, aims of our study were to examine the bleeding and perforation rates of endoscopic polyp resection in patients with large (≥20mm), non- pedunculated polyps and, in particular, to evaluate the influence of size (≥40mm) and age (≥75 years) on the complication rates.

## Material and methods

This multicentre retrospective cohort study was performed between January 2012 and March 2016. For this type of study, formal consent was not required. Patients who had undergone EMR for colorectal non-pedunculated polyps ≥20mm in Leiden University Medical Centre (LUMC) and Isala in Zwolle were included. Pedunculated polyps and malignant appearing polyps which were biopsied and eventually not removed by EMR were excluded in this study. All procedures were performed by endoscopists who were accredited for the Dutch National Bowel Screening Program. In addition, each had performed at least 100 previous EMRs ≥2cm.

### Procedure and equipment

According to the national guideline, anticoagulant therapy (e.g. marcoumar, acenocoumarol) was stopped 3-5 days before the procedure and restarted on the same day after the procedure [36, 37]. The decision to interrupt the use of antiplatelet therapy (e.g. aspirin, clopidogrel, dypiridamol) was evaluated per patient and per type of therapy. Patients using double therapy were instructed to stop one of the antiplatelet drugs before the procedure, in accordance with the guidelines [36–38]. Aspirin could be continued. Double therapy was resumed the day after the procedure. Participants received bowel preparation consisting of picoprep or kleanprep. The mode of sedation was assessed per patient. Most frequently, endoscopic polyp resection was performed under conscious sedation with midazolam and fentanyl. In longer procedures, patients were sedated with propofol and remifentanyl under supervision of an anaesthetic nurse specialist.

Procedures were performed under carbon dioxide insufflation using Olympus CF-HQ190L colonoscopes. The size of the lesion was estimated by the endoscopist during colonoscopy before resection by placing an open biopsy forceps (8mm) next to the lesion. The standard inject-and-cut EMR technique was applied by injecting a solution of Voluven (Hydroxyethyl starch) plasma expander, indigo carmine and in some cases a low concentration of epinephrine (1:100.000) for mucosal lifting. Argon plasma coagulation (pulsed 2, 25W), adrenaline (1:10.000) or clips were used in the case of bleeding. If necessary, e.g. in the case of visible vessels, wounds were approximated by clips. APC was also used to treat residual tissue in case of incomplete resection. All patients were observed for at least one hour. After an uncomplicated procedure they were discharged on the same day, or occasionally after an overnight stay.

### Histology

Adenomas containing >75% villous architecture were defined as villous adenomas and those comprising 25-75% villous architecture as tubulovillous. Focally present high-grade dysplasia (<10%) was considered as low-grade dysplasia.

### Complications

Information on complications was obtained from patients’ electronic patient records including nursing and endoscopy reports. Bleeding was defined as early (<48h after completion of procedure) and delayed (>48h after completion of procedure). Bleeding was registered as a complication when resulting in hospital (re)admission, (re-)intervention and/or therapy (e.g. repeat endoscopy, coiling, blood transfusion or surgery). Clip placement, argon coagulation or adrenaline injection to control bleeding during the initial colonoscopy was not considered as a complication. Perforation was diagnosed either periprocedurally by the endoscopist or by an abdominal CT-scan. Minor damage to the muscle wall, which was managed with clips was not defined as a complication, neither was a (suspicion of) perforation that was directly treated during colonoscopy that did not result in hospital admission. If a complication occurred during the removal of multiple polyps in one session, further investigation was performed to assess which polyp caused the complication (bleeding/perforation).

### Follow-up

In all patients a control visit or telephone appointment was planned about a month after colonoscopy to discuss histopathologic outcomes and follow up. According to the Dutch guidelines for colonoscopy surveillance, the follow-up endoscopy interval depends on the histopathology report (architecture, extent of dysplasia/carcinoma and margin of specimen), the mode of removal (en bloc or piecemeal), the size and number of the adenomas and the location in the colon [39]. If there is uncertainty histologically about the completeness of polyp removal, a surveillance colonoscopy was scheduled 3-6 months later. During follow-up endoscopy, the scar was macroscopically examined and biopsies were taken only in case of a suspected lesion. Residual tissue was treated with cold snare, APC or EMR. In two cases the endoscopy reports and patient records were inconclusive as to whether residual tissue had been detected and were therefore reported as missing and not included in follow-up analysis. In all other cases this was clearly documented.

### Statistical analysis

Data collection and statistical analysis were performed by means of descriptive statistics with IBM SPSS Statistics 23 and Microsoft Office Excel 2010. Fisher’s exact test was used to compare categorical outcome variables. Differences were considered significant if the two-sided *P*-value was <0.05.

## Results

### Study group

EMR of lesions ≥20mm was successfully performed in 343 patients (mean age of 67.4 (SD 8.3), male: *n*=201, (58.6%)). One hundred and three patients (30.1%) used antithrombotic drugs; 15.2% antiplatelet drugs, 11.1% anticoagulants and 3.8% double antiplatelet therapy. In sixty-nine patients, multiple lesions ≥20mm were removed, either in one or more sessions. Table 1 shows an overview of patient and lesion characteristics.

**Table 1:** Patient- and lesion characteristics

	n %
*Patient characteristics*	
Number of patients	343
Mean age (±SD)	67,4 ± 8,3
≥75yr (±SD)	80 (23,3%)
Gender (male)	201 (58,6%)
ASA classification *Missing n=1*	
I	89 (26,0%)
II	228 (66,7%)
III	25 (7,3%)
Antithrombotic therapy *Missing n=1*	103 (30,1%)
Antiplatelet therapy	52 (15,2%)
Anticoagulant therapy	38 (11,1%)
Double therapy	13 (3,8%)
*Lesion characteristics*	
Number of lesions	412
Size	
Mean (mm ±SD)	32,3 ± 13
Median (mm, IQR (25;75))	30,0 IQR (20,0;40,0)
20-40mm	274 (66,5%)
≥40mm	138 (33.5%)
Location	
Ileocecal valve	11 (2,7%)
Cecum	54 (13,1%)
Ascending colon	84 (20,4%)
Hepatic flexure	37 (9,0%)
Transverse colon	51 (12,4%)
Splenic flexure	26 (6,3%)
Descending colon	13 (3,2%)
Sigmoid	55 (13,3%)
Rectosigmoid	15 (3,6%)
Rectum	66 (16,0%)
Paris	
Sessile (0-Is)	205 (50,2%)
Flat (0-IIa, 0-IIb, 0-IIc)	203 (49,8%)
Histology	
Tubulair adenoma	145 (37,2%)
Tubulovillous adenoma	168 (43,1%)
Villous adenoma	10 (2,6%)
Sessile serrated	50 (12,8%)
Other	17 (4,4%)
No dysplasia	52 (12,4%)
Low-grade dysplasia	301 (73,1%)
High-grade dysplasia	30 (7,3%)
Intramucosal carcinoma	11 (2,7%)
Invasive carcinoma	18 (4,4%)

### Lesion characteristics

A total of 412 lesions were reported, of which 138 (33.5%) ≥40mm. The mean size of the resected polyps was 32,3mm (SD 13mm). Two hundred and five (50.2%) were sessile and 203 (49.8%) were flat (missing *n*=4). Most polyps (81.3%) were resected piecemeal.

### Histology

Of all 412 polyps, 145 lesions (37.2%) were tubular adenomas, 158 (43.1%) were tubulovillous adenomas, 10 (2.6%) were villous adenomas and 50 (12.8%) were sessile serrated polyps. In 22 cases, growth patterns were not described in the histology reports. Low grade dysplasia was the most common pathology comprising 301 cases (73.1%). 11 cases (2.7%) showed an intramucosal carcinoma and 18 cases (4.4%) an invasive adenocarcinoma. R0 resection was achieved in 20.8% of all en bloc resections.

### Complications

Table 2 provides an overview of the complications per lesion in this study. Detailed results on complications are presented in Tables 3, 4, 5 and 6.

**Table 2:** Complications and follow-up

	**n (%)**
Number of lesions	412
*Technique*	
En bloc	77 (18,7%)
R0 resection	15 (20,8%)
Piecemeal	335 (81,3%)
*Complications*	
Total bleeding complications	28 (6,8%)
Early bleeding <48h	19 (4,6%)
Delayed bleeding >48h	9 (2,2%)
Perforations	5 (1,2%)
*Surgery*	16 (3,9%)
*Follow-up*	292 (70.9%)
Residual tissue	55 (18.8%)
Enbloc	4 (7.3%)
Piecemeal	51 (92.7%)

**Table 3:** Complications versus polyp size and patients’ age

	20-40mm (*n*=274)	≥40mm (*n*=138)	*p* value	<75jr (*n*=326)*	≥75jr (*n*=86)*	*p* value
Total bleeding complications (*n*=28)	13 (4.7%)	15 (10.9%)	0,036	20 (6.2%)	8 (9,3%)	0,331
Early bleeding (*n*=19)	10 (3,6%)	9 (6,5%)	0,216	14 (4,3%)	5 (5,8%)	0,565
Delayed bleeding (*n*=9)	3 (1.1%)	6 (4.3%)	0,066	6 (1.8%)	3 (3,5%)	0,400
Perforation (*n*=5)	3 (1,1%)	2 (1,4%)	1,000	5 (1,5%)	0	0,588

*evaluated per lesion

**Table 4:** Antithrombotic therapy in relation to polyp size

	20-40mm (*n*=274)	≥40mm (*n*=138)	*p* value
Antithrombotic therapy^*^ (*n*=120)	85 (31,0%)	35 (25,4%)	0,252
No antithrombotic therapy* (*n*=292)	189 (69,0%)	103 (74,6%)	

*evaluated per lesion

**Table 5:** Bleeding complications in antithrombotic therapy

	Antithrombotic therapy^*^ (*n*=120)	No antithrombotic therapy* (*n*=292)	*p* value
Total bleeding complications (*n*=28)	13 (10.8%)	15 (5,8%)	0,051
Early bleeding (*n*=19)	9 (7,5%)	10 (3,4%)	0,117
Delayed bleeding (*n*=9)	4 (3.3%)	5 (1.7%)	0,293

*evaluated per lesion

**Table 6:** Bleeding complications in antithrombotic therapy, subdivided into different therapies

	Antiplatelets (*n*=57)		Coumarins (*n*=47)		Double therapy* (*n*=16)	
	*n*	*p value*	*n*	*p value*	*n*	*p value*
Total bleeding (*n*=13)	4	0,511	4	0,293	5	0,002
Early bleeding (*n*=9)	3	0,454	2	0,677	4	0,004
Delayed bleeding (*n*=4)	1	1,000	2	0,252	1	0,304

*antiplatelet drugs

Total bleeding complication rate was 6.8% (28 cases) and occurred significantly more in polyps ≥40mm compared to polyps 20-40mm (10.9% vs. 4.7%, *p*=0.04). No significant difference was observed in antithrombotic drug use between polyps 20-40mm and ≥40mm (*p*=0.252). Twice as many bleeding complications occurred when using antithrombotic therapy (10.8% vs. 5.1%, *p*=0.051), especially double therapy (31%, *p*=0.002) (Tables 5 and 6). No significant difference was observed in patients <75 years vs. patients ≥75 years (6.2% vs. 9.3%, *p*=0.33). There was one patient who had both an early and delayed bleeding.

*Early bleeding <48h* **:** In 19/412 cases (4.6%) early bleeding was reported. Thirteen of these nineteen cases underwent repeat colonoscopy; four cases needed additional blood transfusion. In the remaining six cases, no colonoscopy was performed. Two of these six patients received a blood transfusion and were sent for angiographic embolisation, the other four were managed conservatively. The mean hospital stay was 2.1 days (range 0-5 days).

There was no significant elevated risk in early bleeding for polyps ≥40mm compared to polyps 20-40mm (6.5% vs. 3.6%; *p*=0.216) and for patients ≥75 years compared to patients <75years (5.8% vs. 4.3%; *p*=0.565). The use of antithrombotics resulted in more early bleeding. However, the difference was not significant (7.5% vs. 3.4%; *p*=0.117) (Table 5), and mainly due to double antithrombotic use (*p*=0.004) (Table 6).

*Delayed bleeding >48h* **.** In 9 of 412 lesions (2.2%) patients were admitted to the hospital for delayed bleeding and all patients underwent repeat colonoscopy. Three cases required blood transfusion. No CT intervention was needed. The mean hospital stay was 1.9 days (range 0-4 days).

Delayed bleeding occurred more in polyps ≥40mm compared to polyps 20-40mm (4.3% vs. 1.1%; *p*=0.066), however this was not significant. No significant difference was found in delayed bleeding complications in patients ≥75 years compared to patients <75 years. (3.5% vs. 1.8%; *p*=0.400). Almost twice as many delayed bleeding complications occurred in patients using antithrombotic drugs compared to patients not using antithrombotic drugs (3.3% vs. 1.7%; *p*=0.293), but this difference was not significant (Table 5).

#### Perforation

Five (1.2%) perforations occurred. One case was managed conservatively, and three cases were successfully closed with clips during the initial endoscopy. Surgical intervention was needed in one case.

No significant difference in perforation rate was observed between resection of 20-40 mm and the resection of polyps larger than 40mm (1.1% vs. 1.4%, *p*=1.000). No perforations occurred in elderly patents above the age of 75 (1.5% vs. 0%, *p*=0.588).

#### Other complications

In total, three complications were observed**.** One patient was admitted for observation after possible aspiration at the end of a colonoscopy under propofol sedation. She was discharged the next day with oral antibiotics without further complications. Another patient was observed after having a post procedural epileptic insult after discharge. Midazolam/fentanyl was used during endoscopy. Lastly, one patient experienced a painless pneumoscrotum directly after the procedure using propofol without further complications..To the best of our knowledge, no cardiovascular events occurred and no deaths related to colonoscopy were observed.

### Surgery

Surgical resection after polypectomy was performed in 16 cases (3.9%). In one patient, surgical intervention was performed because of a perforation during endoscopy. In 15 of these 16 cases, additional surgical resection was performed because of malignant histology of the resected polyp. In five of these fifteen cases, residual carcinoma was found in the surgical specimen. In nine of the fifteen cases, no malignancy was found. One case was lost to follow up as the patient underwent surgery in another hospital.

### Follow-up data

In 292 out of 412 (70.9%) cases, a follow-up colonoscopy was performed with a mean follow-up time of 6.94 months (SD 5.94 months, 95% CI 6.26-7.63 months).

The remaining cases included patients either awaiting a first follow-up procedure (*n*=61), patients without indication for surveillance (e.g. comorbidity, advanced age, or colon resection (*n*=41)), patients lost to follow-up (*n*=9) or patients refusing follow-up (*n*=9).

Assessment of polyp removal sites mostly occurred by macroscopic examination of the scar. Residual tissue/recurrence was found in 55/292 (18.8%) lesions, and was treated with snare or APC. Most residual tissue was found after piecemeal removal, in 51/55 cases (92.7%, *p*<0.05).

## Discussion

The present study supports the premise that EMR of large non-pedunculated polyps is safe in elderly (≥75 years) patients. EMR of giant adenomas (≥40mm) is associated with more bleeding complications but did not lead to more perforations.

Various studies have reported on complication rates after EMR. However, information about the complication rate of EMR of giant polyps and EMR in elderly patients is limited [26, 27, 34, 35]. Our retrospective study evaluated the outcomes and safety of EMR in a large cohort of patients who underwent EMR of polyps ≥20mm. A quarter of the study group were 75 years or older and one third had lesions of more than 40mm.

The overall complication rates observed in our patients were within the range reported in the literature; early bleeding 0-7.9% and delayed bleeding 0-2.3% [11, 14, 19, 27, 28, 34, 40]. We did not observe any deaths due to the interventions. Some studies have reported increased complication rates (bleeding and perforations) of endoscopic removal of larger lesions ≥30mm [12, 23, 26, 35]. The perforation rate of our study in patients with polyps ≥40mm was similar to the perforation rate in patients with polyps of 20-40mm. These findings are in agreement with the study by *Luigiano et al.* [27] On the other hand, we found a significantly higher total bleeding complication rate in polyps larger than 40mm compared to 20-40mm polyps. This finding could not be explained by differences in antithrombotic drug use between both groups. *Sahwney et al.* have also reported lesion size as an independent predictive factor of post polypectomy bleeding [41].

In elderly patients (≥75 years) we did not find significantly more bleeding complications. Also, no perforations were observed in these patients. *Gómez et al.* evaluated the outcomes and safety of colorectal EMR in patients older than 80 years [34]. They reported a total bleeding rate of 2.3%, and a perforation rate of 3%. The authors concluded that EMR for the removal of polyps ≥20mm in elderly patients is safe.

Overall bleeding complications were more frequently observed when antithrombotic drugs were used (borderline significant=0.051), in particular, in patients who used double antiplatelet therapy (*P*<0.05). Guidelines on endoscopy in patients with antithrombotic therapy advise to stop one of antiplatelet drugs when using double therapy 5-7 days before the procedure [36–38]. The other drug can be continued, which is mainly aspirin. Less is known regarding timing of reinitiation of the antiplatelet drug [38]. The drug is restarted the day after the procedure, according to the guidelines [37, 38]. Based on our results we would recommend to consider postponement of restarting the antiplatelet drug after the procedure as we observed significantly more early bleeding complications in patients using double therapy. Numbers are however small and future prospective studies should reveal after how many days the risk of bleeding has reduced after large EMR.

Residual tissue or recurrence was observed in 18.8%, which is within the range reported in literature (4.2-40%) [11, 14, 19, 27, 28, 34]. However, our percentage might be an underestimate because scars were not routinely biopsied. Most residual tissue was found, as expected, after piecemeal removal.

This study adds significantly to the existing literature due to its large size, the high proportion of elderly people and the large number of giant adenomas. A further positive aspect is that we were fully informed about the occurrence of complications after polypectomy. The limitations of our study are its retrospective design and lack of routine biopsies of the polypectomy site at the follow-up colonoscopies.

In conclusion, the implementation of screening programs worldwide has led to the detection of increasing numbers of large non-pedunculated adenomas, often in elderly patients. This number is likely to increase further due to population ageing and higher life expectancy. Since surgical removal of giant adenomas at an advanced age is associated with a substantial mortality (5%), endoscopical removal is increasingly performed [42]. Our study showed that EMR is a safe procedure for both elderly patients above age of 75 and for non-pedunculated colorectal polyps larger than 40mm, although it is associated with significant morbidity, largely due to bleeding. Improved methods are needed to reduce post polypectomy bleeding in patients that use antithrombotic treatments and with giant (≥40mm) polyps.
